# Imminent fracture risk: contributing mechanisms, risk assessment, and implications for treatment

**DOI:** 10.1210/clinem/dgag278

**Published:** 2026-07-14

**Authors:** Tatiane Vilaca, Richard Eastell

**Affiliations:** School of Medicine and Population Health, University of Sheffield, Sheffield S10 2RX, UK; Metabolic Bone Centre, Northern General Hospital, Sheffield S5 7AU, UK; School of Medicine and Population Health, University of Sheffield, Sheffield S10 2RX, UK

**Keywords:** osteoporosis, imminent fracture risk, fracture, fracture risk, secondary fracture prevention

## Abstract

Identifying imminent fracture risk is a critical opportunity to manage osteoporosis. This term refers to the sharply elevated probability of sustaining a subsequent fragility fracture within a determined period—typically 12 to 24 months—after an initial sentinel fracture. While a prior fracture is widely recognized as a primary predictor of future events, epidemiological data show that this risk is not uniform; rather, it peaks significantly within the first 3 to 6 months after injury.

This review discusses a significant clinical gap: traditional assessment tools often focus on long-term, 10-year risk projections, which may delay treatment during this high-vulnerability period. Recognizing imminent risk highlights clinical urgency, indicating the need for early use of therapeutic interventions to rapidly reduce the risk of further fractures. Identifying patients at imminent risk offers an opportunity for secondary prevention, promoting immediate pharmacological action to interrupt the “fracture cascade” and enhance long-term skeletal health.

In this narrative review, we summarize the current literature on imminent fracture risk. We begin by defining imminent fracture risk, then explore its epidemiology and risk factors. We then discuss mechanisms contributing to short-term refracture risk, including falls and acute medical problems (such as stroke) and speculate on the role of potentially accelerated bone loss. As vertebral fractures are often asymptomatic and associated with high imminent fracture risk, we examine some particularities of these fractures. We assess predictive tools and biomarkers including the widely used standard FRAX^©^ and its expanded version FRAXplus^©^. We consider different management strategies and explore research directions and emerging therapies including surrogate mechanisms to assess therapeutic efficacy in future trials and new strategies for the use of new and old drugs.

The concept of “imminent fracture risk” refers to the sharply increased likelihood of sustaining a new fragility fracture within a short, defined period after an initial sentinel event (1-3). Epidemiological studies consistently show that a prior fragility fracture is the strongest predictor of a subsequent fracture; however, the risk does not remain constant over time (4, 5). This high-risk period is generally considered the first 12 to 24 months after the initial fracture, with the greatest absolute increase in risk typically occurring within the first 3 to 6 month (4) (Fig. 1). The concept of imminent fracture risk is important because it requires clinical action and offers a valuable opportunity for prompt intervention (7, 8). Critically, it also influences treatment choices, as the high, imminent risk supports early use of the most effective therapies to reduce fracture risk quickly (7, 8). Lastly, the imminent risk of fractures underscores the limitations of traditional risk assessment tools, as these models commonly focus on long-term risk (eg, 10 years) (9).

**Figure 1:**
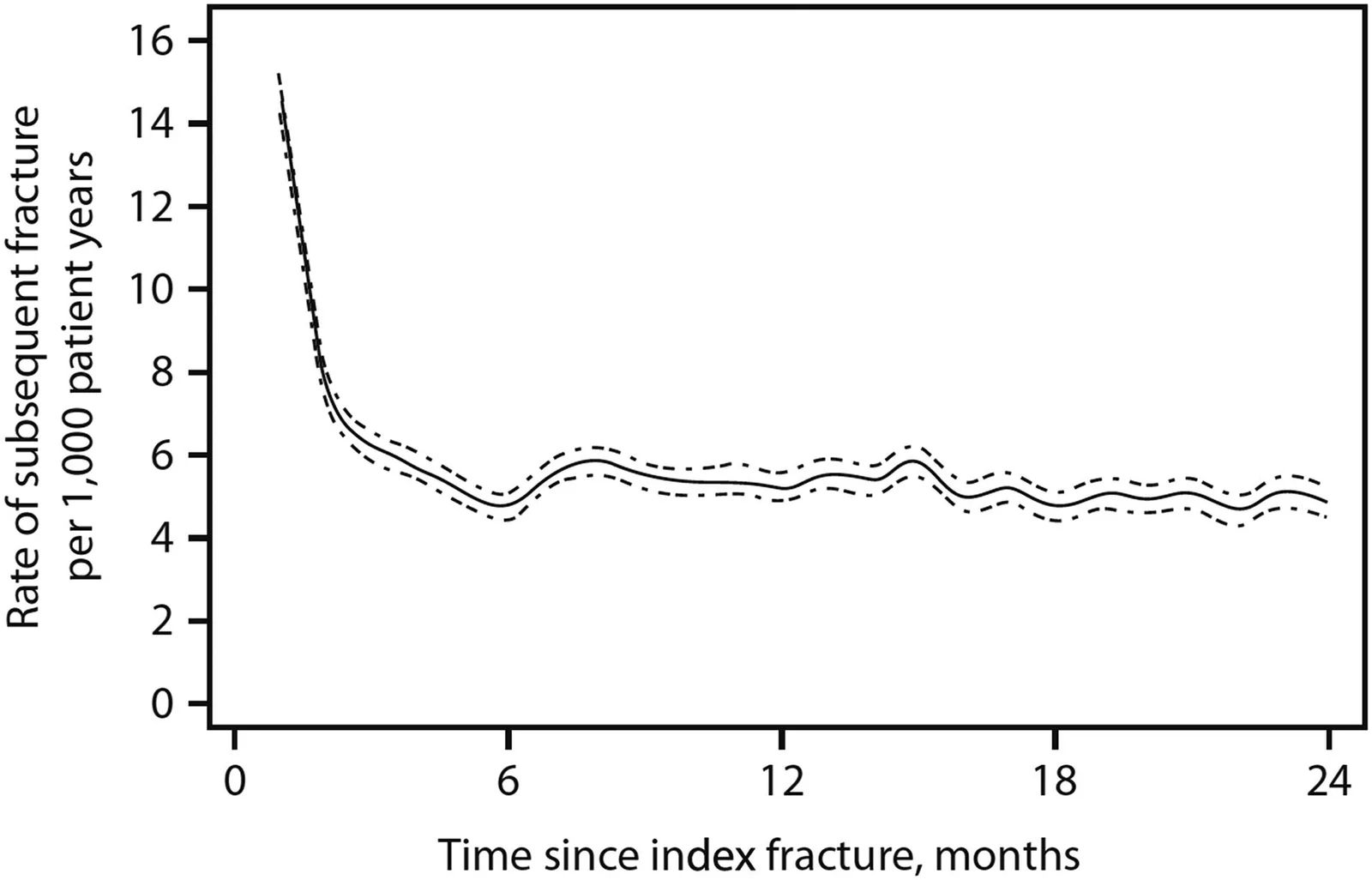
Rate of any subsequent fracture (clinically diagnosed and coded fractures only were counted) during the 24 months following any index fracture on a retrospective, observational cohort study of women aged ≥ 50 years with a fragility fracture identified from Swedish national registers. Reproduced from Banefelt J, et al Risk of imminent fracture following a previous fracture in a Swedish database study. Osteoporos Int 2019; 30(3): 601-9 (6).

## Epidemiology and risk factors for imminent fracture

Robust evidence from several observational studies shows that a prior fragility fracture is a strong predictor of future fractures. Large-scale epidemiological studies consistently demonstrate that the risk of a second fracture varies over time, with several studies indicating a nonlinear pattern (4, 5, 10-12). A retrospective analysis of over 377 000 US Medicare beneficiaries found that among older women who experienced a fracture, 10% had another fracture within one year, 18% within 2 years, and 35% within 5 years (Fig. 2) (12). Similarly, a large Swedish database study of over 242 000 women reported a cumulative incidence of subsequent fracture of 7.1% at 12 months and 12.0% at 24 months (6). In addition, a retrospective population-based analysis from Hong Kong showed an average cumulative imminent risk of fragility fractures of 3.87% in one year and 6.50% at 2 years, and 49.5% of the fractures occurred in the first 2 years (13). Evidence on imminent fracture risk from 64 cohorts was recently summarized in a meta-analysis (14). This pattern has clinical importance; analyses show that a significant proportion of subsequent fractures, ranging from 31% to 45%, occur within the first year after the initial event (5, 10). These consistent findings across populations highlight a high risk of early refracture and emphasize the importance of secondary prevention.

**Figure 2:**
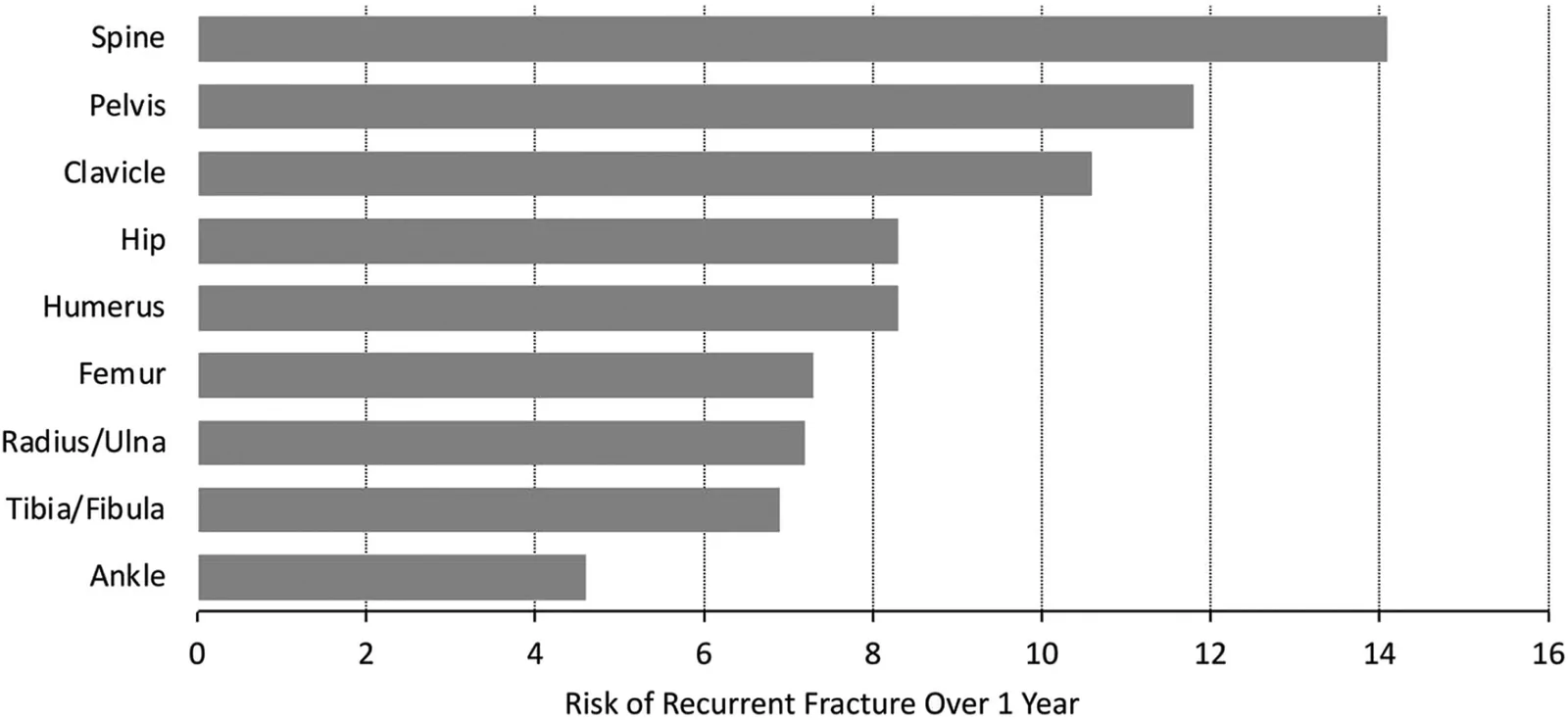
One-year risk of recurrent fracture in women *>*65 years of age, based on site of initial fracture, from Medicare database of 377 561 women with first fracture. Reproduced from Cosman F et al Goal-directed osteoporosis treatment: ASBMR/BHOF task force position statement 2024. J Bone Miner Res 2024; 39(10): 1393-405, adapted from Balasubramanian A. et al Risk of subsequent fracture after prior fracture among older women. Osteoporos Int 2019; 30(1): 79-92.

The increased risk after a fracture is not uniform but follows a distinct temporal pattern. The hazard is highest immediately after the sentinel fracture and declines gradually over time. Data from the Swedish database show that the rate of subsequent osteoporotic fractures peaks in the first month after the initial event (10). A large Icelandic cohort study found that one year after a major osteoporotic fracture, the risk of a second major osteoporotic fracture was 2.7 times higher than in the general population. This relative risk declines over time but remains significant, falling to 1.4 times higher at 10 years (4). Although the most immediate risk period is short, it is important to recognize that the overall fracture risk for these individuals remains considerably higher than baseline for many years (4, 5).

The main factors influencing imminent fracture risk are the characteristics of the sentinel fracture and key patient features. The skeletal site of the initial clinical fracture significantly affected the magnitude of subsequent imminent fracture risk (6, 11, 12). Overall, in older women, the highest imminent risk of a subsequent fracture was associated with initial clinical vertebral fractures, with cumulative incidence rates of 14% at one year and 26% at 2 years among women aged ≥75 years. When assessed using adjusted models (compared to an initial hip fracture), the one-year risk of any subsequent fracture was notably higher after initial clinical vertebral (Odds Ratio [OR] = 1.78), pelvis (OR = 1.40), or clavicle (OR = 1.36) fractures (12). Conversely, initial fractures of the ankle (OR = 0.63) and tibia/fibula (OR = 0.89) were associated with a lower adjusted imminent risk than that of hip fracture. The site of the initial fracture also affected the risk of subsequent hip fracture, with initial clinical vertebral, pelvic, and clavicular fractures all resulting in a one-year subsequent hip fracture risk of ≥3% in women aged 75 or over (12). Notably, advancing age is an independent predictor of imminent risk, with the effect more pronounced in the elderly (4, 12, 15). Female sex is also linked to a significantly higher risk of subsequent fracture (4, 15, 16).

Beyond the sentinel event and demographics, other functional, clinical, and comorbid factors should be assessed (3). A recent history of falls is a key predictor often indicating underlying frailty (15, 17). This frail phenotype is further characterized by reduced physical and cognitive function and poorer general health, which act as both direct and indirect predictors of future fracture (17). The risk is further heightened by specific comorbidities such as cardiovascular disease and chronic obstructive pulmonary disease (COPD), and using medications known to affect bone health or stability, including glucocorticoids and drugs active on the central nervous system (12, 15). Therefore, risk factors for future fractures extend well beyond bone-specific features.

## Mechanisms contributing to imminent fracture risk

### Falls

Several mechanisms contribute to the imminent risk of fractures. The interaction between falls and skeletal fragility is a key factor, as shown by robust replicated evidence (18). A recent fall is a strong predictor of future fracture (19). Analysis of 2 large prospective cohort studies, the Study of Osteoporotic Fractures (SOF) assessing women and the Osteoporotic Fractures in Men Study (MrOS) assessing men, showed that a fall occurring in the past 4 months was significantly linked to a high imminent risk of nonspine and hip fractures in the following year for older women and men. This risk was notably greater than that associated with falls occurring 5-12 months earlier. Quantitatively, a recent fall in older women was associated with an 8.1% absolute risk of nonspine fracture and a 2.5% absolute risk of hip fracture within one year, representing 2.5-fold and 3.1-fold increases in risk, respectively, compared with women who did not fall. Furthermore, this increased imminent fracture risk remained regardless of whether the recent fall resulted in a fracture, confirming that a noninjurious fall is a strong, independent predictor of future fractures (19).

This relationship, however, is not unidirectional. A recent fracture creates a self-perpetuating cycle by significantly increasing the risk of subsequent falls. In the first year after an initial fracture, the relative risk of falling is 2 to 3 times higher than in individuals without a fracture, an effect observed across all age groups and fracture types (20). This vicious cycle, in which falls lead to fractures, which in turn lead to more falls, triggers a cascade of events with potential adverse consequences for the patient.

### Acute medical problems

Acute illness can also influence fracture risk, with a more pronounced risk shortly after an acute event, as shown by replicated observational studies (21, 22). Long-term follow-up found that survivors of acute ischemic stroke had a significantly higher risk of rehospitalization for hip fractures than a nonstroke group, with a 5-year cumulative incidence of 9.5% vs 4.5% (*P* < .0001) (21). This risk was particularly high in the immediate postdischarge period, indicated by an adjusted hazard ratio (HR) of 3.9 (95% CI 2.1−7.3, *P* < .0001) for hip fractures when events were tracked from day 1. Although the hazard decreased over time, the elevated risk remained, falling to an adjusted HR of 2.1 (95% CI 1.5−3.1, *P* < .0001) when observation began on day 31, and further to 1.5 (95% CI 0.9−2.3, *P* = .09) when starting one year after the stroke (21).

### Postfracture morbidity

Furthermore, it is well recognized that the period immediately after any fracture is marked by significant morbidity that extends beyond the acute injury. Fractures are a leading cause of chronic pain, disability, loss of independence, and stiffness, all of which contribute to a substantial decrease in physical activity (19, 23). The subsequent period of immobilization, whether prescribed for healing or self-imposed because of pain and functional limitations, leads to reduced mechanical loading and adverse consequences.

Immobilization following a fracture causes significant localized bone loss. This is evident in the period immediately after an ankle fracture, when bone mineral density (BMD) at the ultradistal ankle can decrease by an average of 12% within just 6 weeks. No change was observed in the femoral neck BMD (24). A similar, localized pattern is seen after a nonweight-bearing distal forearm fracture, resulting in a maximum BMD loss of 9% in the hand by 6 weeks (23). This consistent pattern of bone loss near the fracture site confirms a process referred to as the “regional acceleratory phenomenon”, a local biological response of bone and surrounding tissues to injury (25).

Notably, researchers have reported significant changes in the bone turnover markers following a fracture (23, 24, 26). This elevation directly reflects the different stages of the physiological fracture-healing process and the formation of the fracture callus; it may also indicate a more general change in the skeleton, including accelerated bone loss. Older men who sustain a hip fracture experience accelerated declines at the nonfractured hip that are 2.7 to 5.0 times greater than those observed in age-matched community-dwelling men (27).

### Accelerated bone loss

A mechanistic hypothesis suggests that accelerated bone loss could contribute to imminent fracture risk. Epidemiological data show that higher rates of bone loss are a strong and independent predictor of future fracture risk (28). Similarly, in elderly women, a 5% annual decrease in femoral neck bone mineral density (BMD) is associated with a 40% increase in the relative hazard of fractures, regardless of their initial BMD or age. This suggests that “fast bone losers”—those losing at least 3% to 5% of bone mass annually—face a considerably higher risk than individuals with stable BMD (28).

While some studies show that pre-existing high turnover is an independent risk factor for fractures (28), others challenge the idea that postfracture loss is merely a continuation of a patient's baseline BMD decline (29). Data from a cohort of Caucasian women showed accelerated hip bone loss restricted to the 2-year interval containing the fracture event (29). The data suggest a systemic biological “sprint” triggered by the fracture itself—likely due to inflammation or mechanical unloading—that is temporary rather than a lifelong systemic difference.

### Vertebral fractures

It is important to emphasize some key features of vertebral fractures. While most appendicular fractures result directly from a fall, many osteoporotic vertebral fractures can occur under nontraumatic loads during daily activities (30). Notably, 2-thirds of these fractures may be silent. Unlike fractures at other sites, a common osteoporotic vertebral fracture significantly alters spinal biomechanics, triggering a “cascade” effect that greatly increases the risk of subsequent fracture (30). Finite element analysis demonstrates the profound impact of this structural change; an initial wedge-shaped fracture at the 12th thoracic vertebra (T12) can increase compressive stress on neighboring vertebrae by up to 3.8 times (31) (Fig. 3). Replicated modeling evidence shows that this mechanical overload worsens as the resulting thoracic kyphosis shifts the body's center of gravity forward, further increasing vertebral loads (30, 31). These biomechanical stressors are directly associated with the characteristic bimodal distribution of vertebral fractures, which tend to cluster in the midthoracic and thoracolumbar regions where these forces are most intense (30, 31).

**Figure 3:**
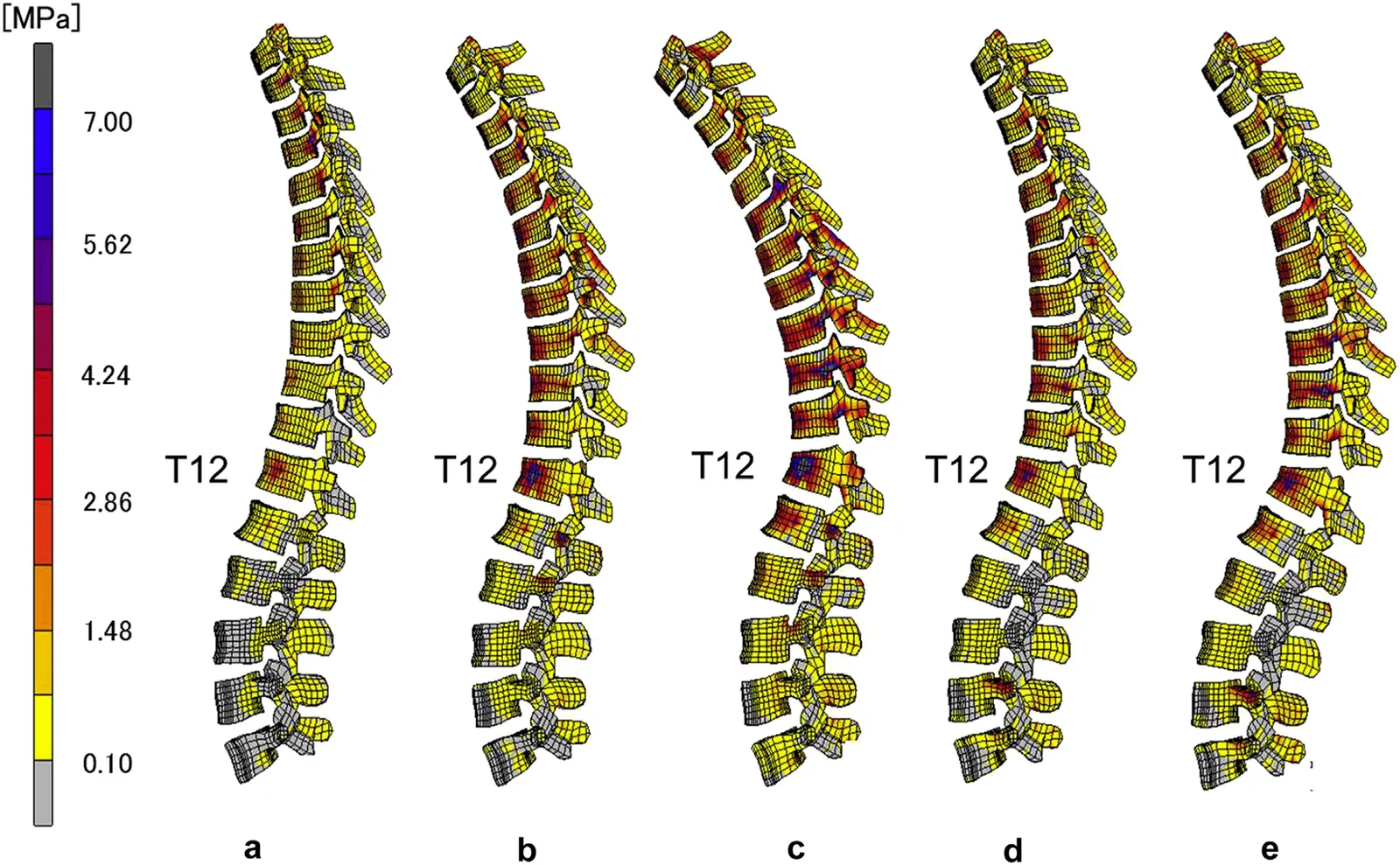
The distributions of compressive principal stress (CPS) values in each model (sagittal view). The CPS values are illustrated in the range of gray (low values), red (medium values), and blue (high values). a–e Correspond to 5 models: (A) a normal model, (B) a 10° kyphosis model, (C) a 20° kyphosis model, (D) a 10° kyphosis model with compensated posture, and (E) a 20° kyphosis model with compensated posture. “Reprinted from The Spine Journal 15 Yoshiyuki Okamoto, Hideki Murakami, Satoru Demura, Satoshi Kato, Katsuhito Yoshioka, Hiroyuki Hayashi, Jiro Sakamoto, Norio Kawahara, Hiroyuki Tsuchiya, The effect of kyphotic deformity because of vertebral fracture: a finite element analysis of a 10° and 20° wedge-shaped vertebral fracture model, 713-720 (2015) with permission from Elsevier.”.

## Predictive tools and biomarkers

### FRAX

The evidence confirming a well-defined period of imminent risk following a sentinel fracture has significant clinical implications. Traditional long-term risk assessment models, such as the widely used standard FRAX® tool, do not account for the recency of a previous fracture and may therefore underestimate short-term risk (9). A meta-analysis of more than 2 000 000 participants from 64 cohorts worldwide showed that the hazard ratio for fractures after a recent event was higher for younger ages and decreased progressively with age and time after a fracture, but there were no differences by fracture site (14). While the relative risk is higher in younger ages (14), the absolute risk of an imminent fracture is higher in the elderly (4, 12, 15). Modifying existing widely accepted tools is a practical strategy for addressing specific predictive gaps without reinventing well-established frameworks (32, 33). Adjustments have been incorporated in the recent update (34), FRAX plus®, which accounts for a sentinel fracture occurring within the last 2 years and its specific anatomical site (32). However, the adjustment algorithms are currently based on data from a single, large Icelandic cohort (4, 9, 32), which raises concerns about wider applicability. For example, in a Swedish cohort, a recent fracture at most sites increased the risk of hip and vertebral fracture (35), as observed in the Icelandic population. In contrast, a large registry-based Canadian study found that recent vertebral and humerus fractures increased near-term risk, but the effect was smaller than previously reported and was not consistent across all fracture sites (36). When assessed, age was an important feature: the increased near-term relative risk in the Canadian cohort was restricted to the younger groups (40-64 years old) (36), while in the Icelandic cohort, probability ratios for fractures were age-dependent and decreased with age (32). Notably, the concurrent risk of death, incorporated into FRAX, is higher in the older and exacerbated after a fracture. This data illustrates the complexity of fracture risk assessment and the challenge of adapting long-term models and has increased interest in developing entirely new tools specifically for short-term predictions.

### Emergent tools for imminent risk

In response to the need for short-term prediction, new algorithms have been developed to estimate imminent (1-year) fracture risk using large-scale administrative data sources (37, 38) and prospective cohort data (39). The Fracture Risk Evaluation Model (FREM) is a notable example, designed to automate 1-year risk prediction using population-level hospital diagnoses (37). The data-driven model's main advantage is its ability to perform automated, population-wide case-finding without requiring laboratory tests or clinical measurements such as BMD. However, external validation has shown that the model systematically overestimated the risk of hip fracture (40). Notably, the model does not account for the recency of the predictor variables, a major flaw for a tool designed to predict imminent risk.

Another example is the model derived from the Frisbee cohort, a prospective cohort of postmenopausal women aged 60 to 85 years (39). Using 5 predictors: BMD, age, comorbidities, falls, and incident central fracture the model uses a nomogram to estimate the imminent risk for fracture, after a recent sentinel event with accuracy comparable to the FRAX^©^ 10-year probability (39) . Risks scores have also been developed to predict 1- and 3-year major osteoporotic and hip fracture risk in Korean women (38). Based on data from more than 1 440 000 women, clinical factors such as body mass index, bone mineral density, fall history within 6 months, prior fractures within 3 years, recent fracture within 1 year and recurrent falls were used to stratify women based on groups: higher scores associated with higher risk of fractures (38). Although several models have been developed the lack of external validation is a challenge for wider use (33).

Nonetheless, the risk of imminent fracture provides a strong rationale for urgent secondary prevention measures. Every fragility fracture should be treated as a critical event that requires prompt clinical review and action to reduce the likelihood of a subsequent, often more serious, fracture.

## Management strategies

Managing imminent fracture risk requires a fundamental shift from traditional long-term (10-year) prevention strategies to an acute, intensive care model. Imminent risk refers to the 12 to 24 months after a sentinel fragility fracture, when the risk of another fracture is highest. This sudden increase in risk is often independent of baseline BMD and requires immediate intervention to target patients during this high-risk period.

Logistically, Fracture Liaison Services (FLS) are essential for the effective delivery of care during the imminent risk window (9). These multidisciplinary, coordinator-led systems are designed to systematically identify and assess fracture patients, ensuring that secondary prevention measures are implemented as soon as possible and no later than 16 weeks after the fracture. FLS models that directly initiate pharmacological treatment, rather than merely providing primary care recommendations, have demonstrated better therapy adherence and a significant reduction in subsequent fracture rate (9).

Effective management must address both nonpharmacological factors and extraskeletal risks, especially falls, which typically trigger most nonvertebral fractures (7, 8). This approach involves ensuring patients are vitamin D-sufficient—usually requiring at least 800 IU of colecalciferol daily—and maintaining an adequate elemental calcium intake of 700 to 1200 mg per day (7, 8, 41). Additionally, all patients should be considered to have a formal falls assessment and be encouraged to participate in at least 12 weeks of progressive resistance and balance training to reduce the bone-muscle loss often seen during postfracture recovery (7, 8, 41).

According to consensus guidelines, individuals who have experienced a vertebral or hip fracture within the past 24 months are deemed at very high risk (7, 8, 42). For these patients, an osteoanabolic-first approach should be considered as the primary pharmacological treatment (7, 8, 42). Practical treatment selection is constrained by drug access, contraindications, and long-term adherence, but head-to-head trials have shown that anabolic agents—such as romosozumab (43) and teriparatide (44)—reduce fracture risk more quickly and to a greater extent than antiresorptives do. Teriparatide and abaloparatide are licensed for use in men and women, but romosozumab is licensed only for women in most countries. Romosozumab, which stimulates bone formation while inhibiting resorption, has demonstrated significant fracture reduction as early as 6 months into treatment (45). Initiating therapy with an anabolic agent maximizes BMD gains, especially at the hip, more effectively than when these agents are used as second- or third-line options (46). Notably, although individuals who sustain a hip fracture are deemed at very high risk, NOGG guidelines recommend treatment with zoledronic acid as the first-line therapy (7). This recommendation is supported by a clinical trial of zoledronic acid in patients with hip fractures, which showed a reduction in clinical fractures and improved survival (47). A key element of the initial anabolic strategy is sequential therapy. Because the duration of anabolic treatment is limited—usually 12 months for romosozumab, 18 months for abaloparatide, and 24 months for teriparatide—it must be immediately followed by a potent antiresorptive agent to maintain and consolidate bone mass (7, 8).

For patients with nonhip, nonvertebral fractures who are not classified as very high risk, the standard clinical priority is to initiate cost-effective antiresorptive therapy (7). Oral bisphosphonates, specifically alendronate and risedronate, are the primary first-line options due to their proven efficacy in reducing fracture risk and their favorable cost-effectiveness profiles. Although index humerus or distal forearm fractures may carry a lower risk than vertebral or hip fractures, they still indicate a significant likelihood of subsequent fractures warranting prompt pharmacological intervention (7).

Alternative treatment pathways are tailored to specific patient demographics and tolerability. For postmenopausal women aged 60 years or younger with a high fracture risk but a low baseline risk of thromboembolic or malignant adverse events, hormone replacement therapy (HRT) is recommended as a viable first-line option (7). When oral bisphosphonates are not tolerated, intravenous zoledronate may be an option. When oral and intravenous bisphosphonates are unsuitable, contraindicated (e.g., in chronic kidney failure), or not tolerated, clinicians should consider other antiresorptive agents, such as denosumab or raloxifene. However, selective estrogen receptor modulators (SERMs), such as raloxifene, primarily prevent vertebral rather than nonvertebral fracture (7, 8). Figure 4 summarizes management recommendations.

**Figure 4:**
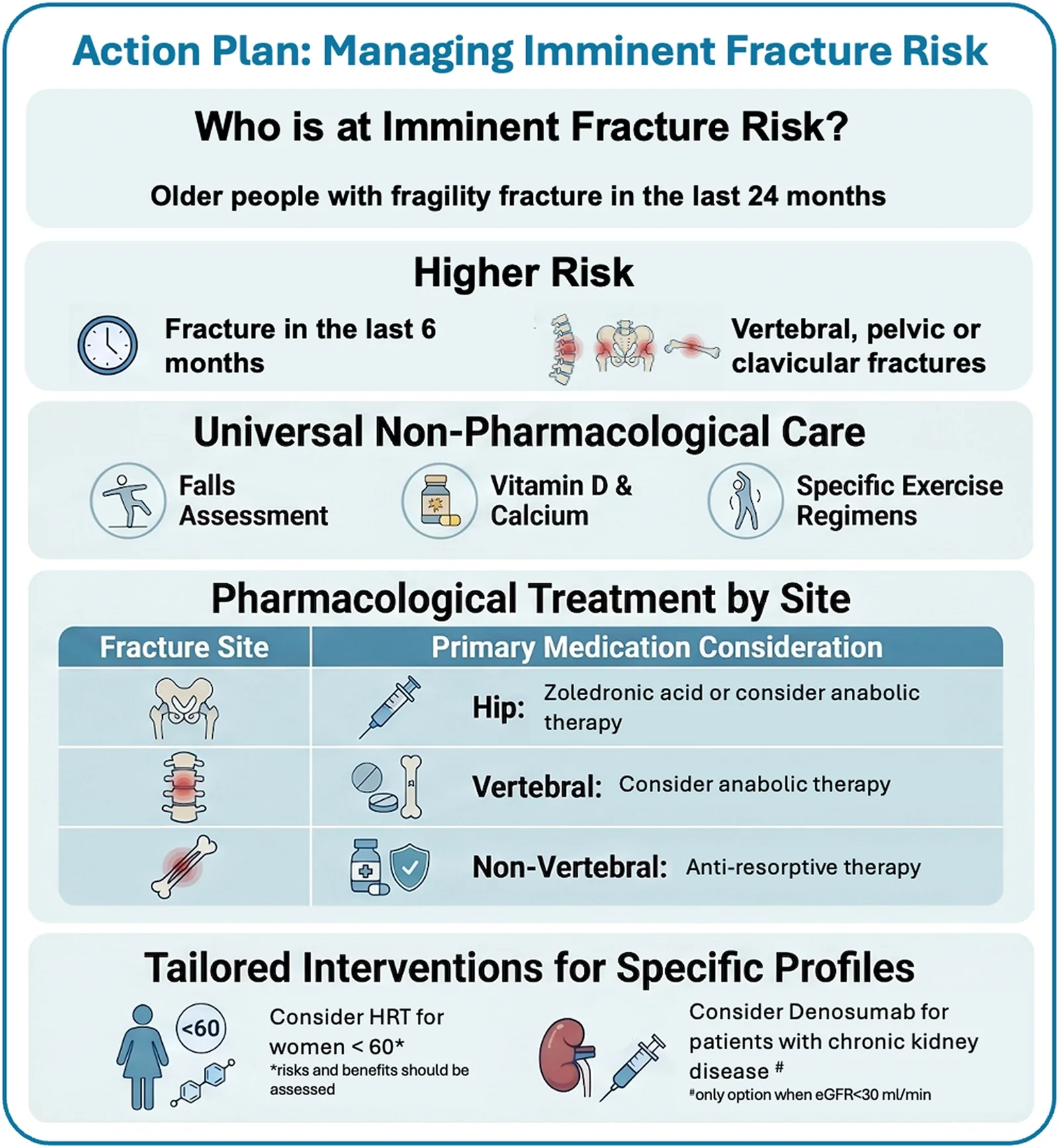
Action plan: managing imminent fracture risk.

Management timelines for these patients require a commitment to long-term therapy, as osteoporosis is a chronic condition that demands lifelong vigilance to prevent future injuries. Current guidelines recommend prescribing oral bisphosphonates for at least 5 years or intravenous zoledronate for 3 years, followed by a formal reassessment of fracture risk (7, 8). After this initial course, a temporary treatment pause, or “bisphosphonate holiday”, lasting 18 to 36 months may be considered for lower-risk patients, provided their clinical profile remains stable, and they are reassessed periodically (7, 8).

Some experts suggest that management should follow a “treat-to-target” framework, with therapy tailored to achieve specific BMD goals (42). The total hip is the preferred skeletal site for monitoring, as it most reliably predicts the risk of both vertebral and nonvertebral fractures. While a T-score of −2.5 is the standard minimum target, higher targets may be recommended in certain situations. Achieving these targets must be confirmed by repeat BMD testing and vertebral imaging to detect asymptomatic incident fractures, which would indicate a need for reassessment (42).

## Emerging therapies or research directions

An acutely increased fracture risk warrants timely and effective interventions. Multiple osteoporosis therapies are available, but new alternatives could broaden the therapeutic arsenal, adding effectiveness, tolerability or convenience and favoring compliance.

### Innovation and research directions

The Study to Advance BMD as a Regulatory Endpoint (SABRE) project (48) represents a milestone in skeletal medicine, culminating in the FDA's decision to qualify total hip BMD as a validated surrogate endpoint for fractures. Historically, the development of new osteoporosis therapies has been hindered by the logistical and ethical challenges of fracture-endpoint trials, which require large patient cohorts and long follow-up periods to achieve statistical significance. By formally recognizing that treatment-induced changes in total hip BMD serve as a reliable proxy for fracture risk reduction, the FDA has created a regulatory path that enables more efficient, cost-effective clinical development, and thereby encourages pharmaceutical innovation.

The SABRE project employed a sophisticated meta-regression analysis of individual patient data from multiple randomized controlled trials on osteoporosis therapies (48). The consortium established that treatment-related changes in total hip BMD at 24 months serve as a robust, statistically significant surrogate for clinical fracture risk reduction (48). This foundational model was subsequently expanded to assess more complex clinical scenarios, including the effectiveness of abbreviated 12- and 18-month measurement intervals (49) and the surrogate's performance across different pharmacological mechanisms of action, ranging from antiresorptive to pure anabolic agents (50). Importantly, the SABRE framework demonstrates high internal validity when applied to active-controlled trials and sequential therapy paradigms (50), indicating that the BMD-as-surrogate model is sufficiently sensitive to capture the cumulative skeletal benefits of “anabolic-first” regimens. The recent approval will undoubtedly promote the development of new antiosteoporosis drugs and contribute to closing the osteoporosis treatment gap.

### Emerging therapies

Pharmacological research is currently focused on dual-action agents and more convenient delivery systems. Romosozumab, which simultaneously increases bone formation and decreases resorption, has paved the way for pipeline drugs such as AGA2118, a bispecific antibody targeting sclerostin and Dickkopf1, currently in Phase II trials that show promise for rapid BMD accrual (51). Additionally, the emergence of oral anabolic therapies (eg, EB613) (52) and biosimilar denosumab (53) is addressing challenges related to patient compliance and costs. These additional medicines will expand therapeutic options for osteoporosis.

## Conclusion

Imminent fracture risk is not an elevated long-term risk when viewed up close, but an acute, transient spike in risk immediately after a fracture. Evidence confirms that the first 12 to 24 months following a sentinel fragility fracture—specifically the first 3 to 6 months—represent a period of maximal vulnerability during which the probability of a subsequent fracture is sharply elevated. This risk is not static; it is significantly influenced by the site of the initial fracture, with vertebral, pelvic, and clavicular events carrying higher odds of recurrence than hip fractures. Notably, the patients with imminent fracture risk form a heterogeneous population in which falls and comorbidity-related risks play an important role, beyond bone features. Traditional 10-year risk models often fail to capture this transient window, but new updates can account for the acute risk increase. Recognizing this sharp increase in risk is crucial, as it requires immediate intervention to prevent recurrence and mitigate substantial morbidity and mortality.

Despite raising awareness of the acute increase in the risk of a subsequent fracture after a sentinel event, we recognize that most evidence comes from cohort studies in Europe and North America, as well as modeling studies that suggest associated mechanisms. In addition, no randomized controlled trial has investigated the effect of therapies specifically in the context of imminent fracture risk.

Future directions must focus on integrating imminent risk parameters into standard assessment tools and clinical guidelines to overcome the limitations of the current approach. Validation in different populations is also required. Research should prioritize refining dynamic risk models that incorporate features of the recent fracture and key patient variables, such as recent falls, to better guide intervention. Randomized controlled trials in the context of imminent fracture risk would provide valuable information. Furthermore, prospective studies are needed to evaluate the long-term benefits of prioritizing rapid-acting, high-efficacy therapies immediately after a sentinel event, as the magnitude of the benefit remains uncertain. By narrowing the gap between fracture occurrence and timely treatment, the health care community can more effectively reduce the high morbidity associated with recurrent fragility fractures.

## Data Availability

No new data were generated or analysed in support of this research.
